# Ameliorative effect of nanocurcumin and *Saccharomyces* cell wall alone and in combination against aflatoxicosis in broilers

**DOI:** 10.1186/s12917-022-03256-x

**Published:** 2022-05-14

**Authors:** Aya Ashry, Nabil M. Taha, Mohamed A. Lebda, Walied Abdo, Eman M. El-Diasty, Sabreen E. Fadl, Mohamed Morsi Elkamshishi

**Affiliations:** 1 Biochemistry Dept., Faculty of Veterinary Medicine, Matrouh University, Matrouh, Egypt; 2 Department of Biochemistry, Faculty of Veterinary Medicine, Alexandria University, Alexandria, 21526 Egypt; 3 Pathology Department, Faculty of Veterinary Medicine, Kafrelsheikh University, Kafr El-Sheikh, 33516 Egypt; 4 Mycology and Mycotoxins Department, Animal Health Research Institute (ARC), Dokki, Egypt; 5 Department of Animal Hygiene and Zoonoses, Faculty of Veterinary Medicine, Matrouh University, Matrouh, Egypt

**Keywords:** Aflatoxin, *Saccharomyces* cell wall, Nanocurcumin, Biochemistry, Pathology, Liver gene

## Abstract

**Background:**

The adverse effect of aflatoxin in broilers is well known. However, dietary supplementation of *Saccharomyces* cell wall and/or Nanocurcumin may decrease the negative effect of aflatoxin B1 because of the bio-adsorbing feature of the functional ingredients in Yeast Cell Wall and the detoxification effect of curcumin nanoparticles. The goal of this study was to see how *Saccharomyces* cell wall/Nanocurcumin alone or in combination with the aflatoxin-contaminated diet ameliorated the toxic effects of aflatoxin B1 on broiler development, blood and serum parameters, carcass traits, histology, immune histochemistry, liver gene expression, and aflatoxin residue in the liver and muscle tissue of broilers for 35 days. Moreover, the withdrawal time of aflatoxin was measured after feeding the aflatoxicated group an aflatoxin-free diet. Broiler chicks one day old were distributed into five groups according to *Saccharomyces* cell wall and/or nanocurcumin with aflatoxin supplementation. The G1 group was given a formulated diet without any supplements. The G2 group was supplemented with aflatoxin (0.25 mg/kg diet) in the formulated diet. The G3 group was supplemented with aflatoxin (0.25 mg/kg diet) and *Saccharomyces* cell wall (1 kg/ton diet) in the formulated diet. The G4 group was supplemented with aflatoxin (0.25 mg/kg diet) and nanocurcumin (400 mg/kg) in the formulated diet. The G5 group was supplemented with aflatoxin (0.25 mg/kg diet) and *Saccharomyces* cell wall (1 kg/ton diet) in combination with nanocurcumin (200 mg/kg) in the formulated diet.

**Results:**

According to the results of this study, aflatoxin supplementation had a detrimental impact on the growth performance, blood and serum parameters, carcass traits, and aflatoxin residue in the liver and muscle tissue of broilers. In addition, aflatoxin supplementation led to a liver injury that was indicated by serum biochemistry and pathological lesions in the liver tissue. Moreover, the shortening of villi length in aflatoxicated birds resulted in a decrease in both the crypt depth ratio and the villi length ratio. The expression of *CYP1A1* and *Nrf2* genes in the liver tissue increased and decreased, respectively, in the aflatoxicated group. In addition, the aflatoxin residue was significantly (*P* ≤ 0.05) decreased in the liver tissue of the aflatoxicated group after 2 weeks from the end of the experiment.

**Conclusion:**

*Saccharomyces* cell wall alone or with nanocurcumin attenuated these negative effects and anomalies and improved all of the above-mentioned metrics.

**Supplementary Information:**

The online version contains supplementary material available at 10.1186/s12917-022-03256-x.

## Background

Aflatoxins are secondary metabolites of mycotoxins that are naturally occurring and derived from *Aspergillus flavus* and *Aspergillus parasiticus*. It’s known to have very toxic and carcinogenic impacts. Among the different serious aflatoxins (B1, B2, G1, and G2, M1, M2), aflatoxin B1 (AFB1) is the most harmful one. It is also considered a Group I carcinogen. Significant economic losses are posed due to human and animal consumption of food or feed contaminated by AFB1 as its administration exhibits harmful hepatotoxic, teratogenic, mutagenic, and carcinogenic effects on humans and many species of livestock [1]. The most important economic effect of aflatoxicosis in broilers is the decrease in body weight gain, probably caused by disturbances in protein metabolism [2, 3]. On the other hand, aflatoxin has a bad effect on relative organ weight [4, 5], especially on the liver tissue [6, 7]. Moreover, aflatoxin affects hematological [8, 9] and biochemical [10] parameters. In addition, Zaker**-**Esteghamati et al. [11] reported the negative effects of aflatoxin on growth performance, carcass and meat characteristics, and intestinal microflora.

Curcumin, or diferuloylmethane, is a naturally occurring low molecular weight polyphenol, which is considered the active principle substance of the plant Curcuma longa (turmeric’s rhizome) [12]. Previous studies have proven that curcumin can prevent the hepatotoxic and carcinogenic effects of AFB1 in rats [13]. Moreover, Emadi et al. [14] reported that curcumin is a natural antioxidant that protects the liver from acetaminophen-induced damage in Japanese quail. Curcumin can inhibit liver CYP450 isozymes, so it could be a potentially favorable chemopreventive agent for many carcinogenic and toxic agents like AFB1 [1]. But the low oral bioavailability of curcumin due to low absorption, fast metabolism, and fast systemic elimination from the body has been a major problem [15]. Thus, Sayrafi et al. [16] and Heidary et al. [17] said that utilizing curcumin nanoparticles improves its bioavailability in the body while increasing its solubility in water. Curcumin nanoparticle formulations cause a 10–14-fold higher absorption rate compared with the same oral dose of free curcumin [18].

On the other side, prebiotics are one of the most widely used components in production farms as functional foods today [19]. Yeast (*Saccharomyces cerevisiae*) cell wall (YCW) as a feed additive containing two main prebiotic components (mannan oligosaccharides and 1,3/1,6 β-glucans) has been used in poultry production, improving performance by increasing the average daily gain, improving the feed conversion, and enhancing immunological response [20]. Supplementing diets with yeast cell walls increases weight gains and improves feed conversion [20]. A trial of using a cell wall of yeast to minimize the adverse effects of aflatoxin-contaminated diets in broiler chicks resulted in a significant increase in BW gain and improved feed consumption [21].

This work was carried out to investigate the effects of dietary supplemental *Saccharomyces* cell wall extract and/or nanocurcumin on the growth performance, some carcass traits, hematological parameters, some blood biochemical changes, some gene expressions, and some organ histopathology of broiler chicks fed on an aflatoxin containing diet.

## Results

### Clinical signs and postmortem lesions of the aflatoxicated broilers

After two weeks from the beginning of the trial, clinical symptoms of the aflatoxicated broilers were observed. The observed clinical symptoms are retarded growth, leg paralysis and lameness, wing paralysis, whitish diarrhea, and eye lesions. There was no mortality in the aflatoxicated broilers during this trial. At the end of the trial, the euthanized aflatoxicated broilers showed an enlarged liver and kidney with generalized congestion in the muscle tissue. On the other side, the addition of *Saccharomyces* cell wall/Nanocurcumin alone/in combination to the aflatoxin-contaminated diet alleviated the toxic effects of aflatoxin B1 on internal organs in broilers fed on for 35 days. These findings were indicated by the normal appearance of the internal organs and muscles.

### Growth performance

The effect of the addition of *Saccharomyces* cell wall/Nanocurcumin alone/in combination to the aflatoxin-contaminated diet against the toxic effects of aflatoxin B1 on growth performance in broilers fed on for 35 days has been shown in Table 1. The mean values of final body weight, BWG, and feed intake in the aflatoxin-fed group (G2) were significantly (*P* ≤ 0.05) decreased than that of the control group (G1). Dietary supplementation of nanocurcumin and/or *Saccharomyces* cell wall lowered the negative effect of aflatoxin on the final body weight, BWG, and feed intake of broilers. As shown in the table, the combination of nanocurcumin and *Saccharomyces* cell wall had the highest effect, except in the case of feed intake, both the combination of nanocurcumin and *Saccharomyces* cell wall as in (G5) and the supplementation of *Saccharomyces* cell wall only as in (G3) showed the same effect. On the other hand, the mean value of FCR in the aflatoxin-fed group (G2) was significantly (*P* ≤ 0.05) increased compared to other groups.

**Table 1:** Effect of dietary Nano curcumin and *Saccharomyces* cell wall on the performance values for broiler chicks fed on diet containing 0.25 mg aflatoxin B1 / kg diet at 1 to 35 day

Groups Items	**G1**	**G2**	**G3**	**G4**	**G5**
Initial weight (g)	55 ± 1.50	57 ± 1.50	57 ± 0.90	56 ± 1.60	55.4 ± 1.11
Final weight (g)	2385 ± 3.495^a^	1605 ± 2.79^e^	2038 ± 3.81^d^	2097 ± 2.91^c^	2110 ± 2.24^b^
BWG (g)	2329.80 ± 4.13^a^	1548.00 ± 3.26^e^	1980.60 ± 4.12^d^	2042.30 ± 3.72^c^	2054.60 ± 2.63^b^
Feed intake (g)	3460 ± 0.58^a^	2800 ± 0.00^d^	3100 ± 0.58^b^	3089 ± 0.57^c^	3100 ± 0.00^b^
FCR	1.5 ± 0.011^c^	1.81 ± 0.004^a^	1.57 ± 0.004^b^	1.51 ± 0.002^c^	1.51 ± 0.003^c^

Values are means ± standard error

Mean values with different subscript letters (a-e) at the same row significantly differ at (*P* ≤ 0.05). G1 = group fed control diet without supplement; G2 = group fed control diet with aflatoxin; G3 = group fed control diet with aflatoxin and *Saccharomyces* cell wall; G4 = group fed control diet with Nano curcumin; G5 = group fed control diet with *Saccharomyces* cell wall and Nano curcumin

### Carcass traits

The effect of the addition of *Saccharomyces* cell wall/Nanocurcumin alone/in combination to the aflatoxin-contaminated diet against the toxic effects of aflatoxin B1 on some carcass traits in broilers fed on for 35 days as a percent of live body weight is shown in Table 2. There was a significant (*P* ≤ 0.05) decrease in the dressing percentage and leg muscle in the G2 when compared with the other groups. Moreover, there was a significant (*P* ≤ 0.05) increase in dressing percentage in the G5 when compared with the G2 and G3. On the other side, there was an insignificant (*P* ≤ 0.05) increase in the dressing percentage in G5 when compared with G1 and G4. Regarding the result of the breast muscle, there was a significant (*P* ≤ 0.05) increase in the G5 when compared with G1 and G2 (control negative and control positive groups), but non-significant (*P* ≤ 0.05) when compared to G3 and G4. Moreover, the relative weight of the liver in G2 showed a significant (*P* ≤ 0.05) increase when compared with the other groups, and there was an insignificant (*P* ≤ 0.05) decrease between G1 and G4, G3, and G5. Meanwhile, thymus relative weight showed a significant (*P* ≤ 0.05) increase in the G3 when compared with the G1 but was non-significant (*P* ≤ 0.05) when compared with the other groups. Regarding the result of the relative organ weight of the spleen, there was a significant (*P* ≤ 0.05) decrease and an insignificant increase in G1 compared to G2 and the other remaining groups, respectively. On the other hand, the relative weight of proventriculus and bursa was insignificantly (*P* ≤ 0.05) increased in G2 when compared with the other groups, while there was an insignificant (*P* ≤ 0.05) increase in the relative weight of gizzard in G5 when compared with the other groups.

**Table 2:** Effect of dietary Nano curcumin and *Saccharomyces* cell wall on some carcass traits values for broiler chicks fed on diet containing 0.25 mg aflatoxin B1 / kg diet at 1 to 35 day

Groups Items %	**G1**	**G2**	**G3**	**G4**	**G5**
Dressing	76.69 ± 3.48 ^ab^	68.46 ± 2.42 ^c^	73.25 ± 0.31 ^b^	76.45 ± 0.69 ^ab^	80.52 ± 1.32^a^
Breast muscle	45.32 ± 2.48 ^b^	43.59 ± 0.17 ^b^	46.54 ± 1.29^ab^	46.78 ± 1.24^ab^	50.11 ± 1.23^a^
Leg muscle	31.37 ± 1.01^a^	24.78 ± 0.68^b^	30.41 ± 0.63^a^	29.67 ± 0.56^a^	29.66 ± 0.38^a^
Liver	2.19 ± 0.12^b^	3.94 ± 0.87^a^	1.57 ± 0.19^b^	1.86 ± 0.09^b^	1.53 ± 0.05^b^
Gizzard	1.50 ± 0.22	1.41 ± 0.09	1.58 ± 0.13	1.60 ± 0.08	1.33 ± 0.13
Provetriculus	0.26 ± 0.01	0.27 ± 0.03	0.26 ± 0.03	0.27 ± 0.02	0.27 ± 0.01
Thymus	0.10 ± 0.02^b^	0.17 ± 0.05^ab^	0.19 ± 0.02^a^	0.11 ± 0.02^ab^	0.12 ± 0.02^ab^
Spleen	0.09 ± 0.03^ab^	0.15 ± 0.03^a^	0.05 ± 0.00^b^	0.06 ± 0.01^b^	0.04 ± 0.00^b^
Bursa	0.12 ± 0.02	0.19 ± 0.07	0.09 ± 0.01	0.09 ± 0.00	0.09 ± 0.02

Values are means ± standard error

Mean values with different subscript letters (a-b) at the same row significantly differ at (*P* ≤ 0.05). G1 = group fed control diet without supplement; G2 = group fed control diet with aflatoxin; G3 = group fed control diet with aflatoxin and saccharomyces cell wall; G4 = group fed control diet with Nano curcumin; G5 = group fed control diet with saccharomyces cell wall and Nano curcumin

### Hematological parameters

The effect of the addition of *Saccharomyces* cell wall/Nanocurcumin alone/in combination to the aflatoxin-contaminated diet against the toxic effects of aflatoxin B1 on hematological parameters in broilers fed on for 35 days is shown in Table 3. There was a significant (*P* ≤ 0.05) decrease in RBCs count, HCT, WBCs count, and LYM in the G2 when compared with the other groups. On the other side, there was an insignificant (*P* ≤ 0.05) decrease in Hb concentration in the G2 compared with the other groups. Regarding the result of HCT (%), there was an insignificant (*P* ≤ 0.05) decrease and increase in the HCT (%) in G3 when compared with G1, G5, and G4, respectively. Meanwhile, HET, ESI, and MON counts showed a significant (*P* ≤ 0.05) increase in G2 when compared with the other groups.

**Table 3:** Effect of dietary Nano curcumin and *Saccharomyces* cell wall on hematological values for broiler chicks fed on diet containing 0.25 mg aflatoxin B1 / kg diet at 1 to 35 day

Groups Items %	**G1**	**G2**	**G3**	**G4**	**G5**
RBCs count (× 10^6^/µl)	3.57 ± 0.17 ^a^	2.30 ± 0.18 ^c^	3.30 ± 0.10^ab^	3.13 ± 0.04^b^	3.43 ± 0.15^ab^
Hb (g/dl)	11.07 ± 0.75	9.36 ± 0.85	9.89 ± 0.29	9.38 ± 0.11	10.29 ± 0.44
HCT (%)	41.92 ± 1.06^a^	29.55 ± 0.48^c^	39.55 ± 1.15^ab^	37.52 ± 0.44^b^	41.16 ± 1.78^a^
WBCs count (× 10^3^/µl)	20.12 ± 1.00 ^a^	13.57 ± 1.31 ^b^	16.52 ± 0.89 ^ab^	18.46 ± 1.72 ^a^	18.63 ± 1.09 ^a^
LYM (× 10^3^/µl)	62.49 ± 1.86 ^a^	40.25 ± 1.08 ^b^	55.78 ± 2.56 ^a^	59.14 ± 3.95 ^a^	59.81 ± 3.75 ^a^
HET (× 10^3^/µl)	32.76 ± 1.75^b^	50.70 ± 0.92^a^	37.63 ± 2.01^b^	35.71 ± 3.48^b^	35.80 ± 3.90^b^
ESI (× 10^3^/µl)	1.17 ± 0.09^c^	2.14 ± 0.09^a^	1.54 ± 0.03^b^	1.27 ± 0.09^c^	1.15 ± 0.02^c^
MON (× 10^3^/µl)	3.59 ± 0.28 ^c^	6.92 ± 0.42 ^a^	5.05 ± 0.55 ^b^	3.87 ± 0.42 ^bc^	3.24 ± 0.21 ^c^

Values are means ± standard error

Mean values with different subscript letters (a-c) at the same row significantly differ at (*P* ≤ 0.05). G1 = group fed control diet without supplement; G2 = group fed control diet with aflatoxin; G3 = group fed control diet with aflatoxin and saccharomyces cell wall; G4 = group fed control diet with Nano curcumin; G5 = group fed control diet with saccharomyces cell wall and Nano curcumin

*RBCs* Red blood cells, *Hb* Hemoglobin, *HCT* Hematocrit, *WBCs* White blood cells, *LYM* Lymphocyte, *HET* Heterophil, *ESI* Eosinophil, *MON* Monocytes, *BAS* Basophil

### Biochemical parameters

The effect of the addition of *Saccharomyces* cell wall/Nanocurcumin alone/in combination to the aflatoxin-contaminated diet against the toxic effects of aflatoxin B1 on some biochemical parameters in broilers fed on for 35 days is shown in Table 4. There was a significant (*P* ≤ 0.05) increase in the ALT, AST, and ALP activities in the G2 (control positive) when compared with the other groups. On the other hand, there was a significant (*P* ≤ 0.05) decrease in the ALT activity in the G4 and G5 when compared with the G1 group (control negative). Meanwhile, AST and ALP activities were significantly (*P* ≤ 0.05) increased in the G4 and G5 when compared with the G1 group. Regarding the results of total protein, albumin, and globulin, there were significant (*P* ≤ 0.05) decreases in all of these parameters in the G2 when compared with the G1. On the other side, there were significant and insignificant (*P* ≤ 0.05) increases in the total protein and globulin and albumin, respectively, in the G4 and G5 when compared with the G1. Regarding the results of the kidney function, the concentrations of urea and uric acids were insignificantly (*P* ≤ 0.05) increased in G2 when compared with the other groups. On the other side, the creatinine concentration was significantly (*P* ≤ 0.05) increased in G2 when compared with G1 but was non-significant (*P* ≤ 0.05) when compared with the other groups.

**Table 4:** Effect of dietary Nano curcumin and *Saccharomyces* cell wall on some biochemical values for broiler chicks fed on diet containing 0.25 mg aflatoxin B1 / kg diet at 1 to 35 day

Groups Items	**G1**	**G2**	**G3**	**G4**	**G5**
ALT (U/L)	25.30 ± 2.48 ^c^	39.00 ± 2.08 ^a^	31.37 ± 0.58 ^b^	19.00 ± 2.31 ^d^	16.60 ± 0.17^d^
AST (U/L)	20.00 ± 1.73 ^e^	138.33 ± 2.03 ^a^	66.67 ± 2.19^b^	36.10 ± 0.84^d^	58.50 ± 0.87^c^
ALP (U/L)	931.67 ± 2.73^b^	1197.33 ± 1.76^a^	911.33 ± 1.86^c^	518.00 ± 3.61^e^	537.00 ± 1.53^d^
T. Protein (g/dl)	6.07 ± 0.12^b^	3.60 ± 0.21^c^	6.33 ± 0.12^b^	7.27 ± 0.19^a^	7.03 ± 0.15^a^
Albumin (g/dl)	3.60 ± 0.18 ^a^	2.81 ± 0.41 ^b^	3.54 ± 0.04 ^a^	3.71 ± 0.12 ^a^	3.81 ± 0.03 ^a^
Globulin (g/dl)	2.47 ± 0.06^d^	0.79 ± 0.08^e^	2.79 ± 0.08^c^	3.55 ± 0.07 ^a^	3.23 ± 0.12^b^
Creatinine (mg/dl)	0.32 ± 0.01^b^	0.44 ± 0.03^a^	0.38 ± 0.02^ab^	0.38 ± 0.02^ab^	0.38 ± 0.04^ab^
Urea (mg/dl)	11.31 ± 0.66	12.23 ± 0.39	11.44 ± 0.68	11.55 ± 0.16	12.05 ± 0.65
Uric acid (mg/dl)	5.69 ± 0.46	6.92 ± 0.92	5.51 ± 0.51	5.76 ± 0.53	6.23 ± 0.77

Values are means ± standard error

Mean values with different subscript letters (a-e) at the same row significantly differ at (*P* ≤ 0.05). G1 = group fed control diet without supplement; G2 = group fed control diet with aflatoxin; G3 = group fed control diet with aflatoxin and saccharomyces cell wall; G4 = group fed control diet with Nano curcumin; G5 = group fed control diet with saccharomyces cell wall and Nano curcumin

### Histopathology of the liver and intestine

**Fig. 1:**
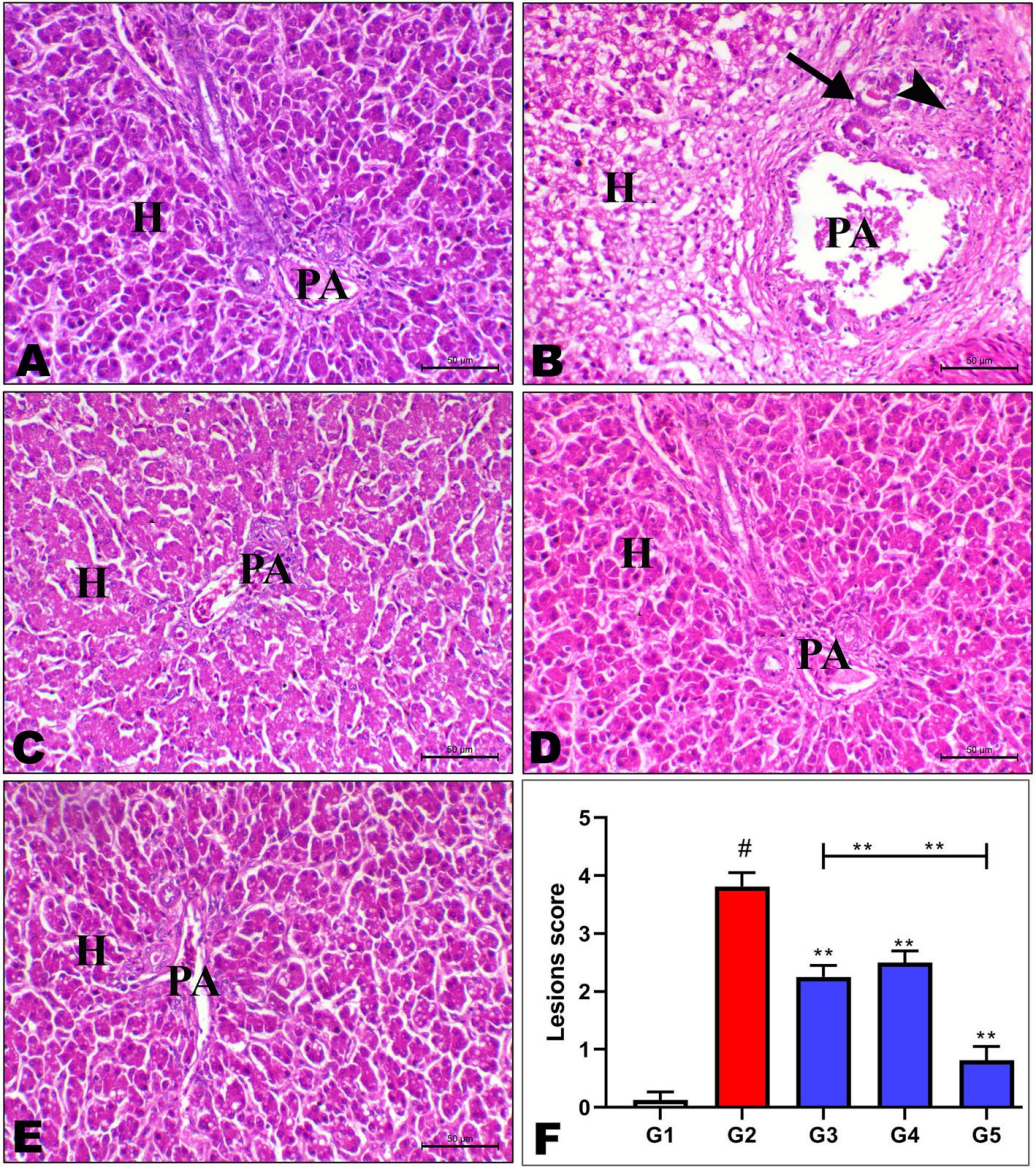
Effect of dietary Nano curcumin and Saccharomyces cell wall on photomicrograph of hepatic tissues for broiler chicks fed on diet containing 0.25 mg aflatoxin B1 / kg diet at 1 to 35 day stained with hematoxylin and eosin (**H**&**E**) where (**A**) G1: showing normal histology in which the hepatocytes (**H**) and portal area (PA) consisting of bile duct, portal vein, hepatic arteriole brunch, (**B**) G2: showing fibrosis of the portal area (PA) with newly formed bile ducts (arrow) and mononuclear cells infiltration within the portal area (arrow head) beside wide periportal hepatocytic cells necrosis (**H**), (**C**) G3: showing the hepatocytes (H) with multifocal mild cytoplasmic sharp outlines vacuoles referring to fatty change and normal portal area (PA), (**D**) G5: showing normal histology including the hepatocytes (H) and portal area (PA) consisting of bile duct, portal vein, hepatic arteriole brunch, (**E**) G4: showing normal hepatocytes (H) and portal area (PA) slightly infiltrated with mononuclear cells, (**F**) showing quantities scoring of the hepatic lesions in different groups, bar = 50 µm

Histopathology of the hepatic tissues of different treated groups was illustrated in Fig. 1, which fed on the aflatoxin-free diet, aflatoxin diet, and aflatoxin diet with *Saccharomyces* cell wall/nanocurcumin alone/in combination. It was observed that the broiler chicks group that was fed an aflatoxin-free diet showed normal histology in which the normal hepatocytes (H) and around the portal area (PA) consisted of the bile duct, portal vein, and hepatic arteriole branch (Fig. 1A). Conversely, the addition of aflatoxin had an adverse effect on liver hepatobiliary tissue histopathology where a photomicrograph of the liver of the aflatoxin group showed marked periportal hepatocytic cells necrosis in addition to mononuclear cells infiltration, fibrosis of the portal area, and regenerative (PA) with newly formed bile ducts and mononuclear cells infiltration within the portal area beside wide periportal hepatocytic cells necrosis (Fig. 1B). Meanwhile, the photomicrograph of the livers of broiler chicks fed aflatoxin with *Saccharomyces* cell wall alone (G3) showed a decrease in the periportal hepatic necrosis, normal hepatocytes (H), and portal area (PA) with slight infiltration of mononuclear cells (Fig. 1C). On the other side, the photomicrograph of the livers of the aflatoxin with Nano alone group (G4) showed the hepatocytes (H) with multifocal mild cytoplasmic sharp outlines vacuoles referring to fatty change and normal portal area (PA) (Fig. 1D). Meanwhile, a photomicrograph of the liver of the aflatoxin with two treatments in combination (G5) group showed normal histology including the hepatocytes (H) and portal area (PA), which consisted of the bile duct, portal vein, and hepatic arteriole brunch (Fig. 1E). Quantities scoring of the hepatic lesions showed a marked increase in the lesions score within G2, and a decrease in G3 and G4, with a marked decrease in G5 (Fig. 1F).

**Fig. 2:**
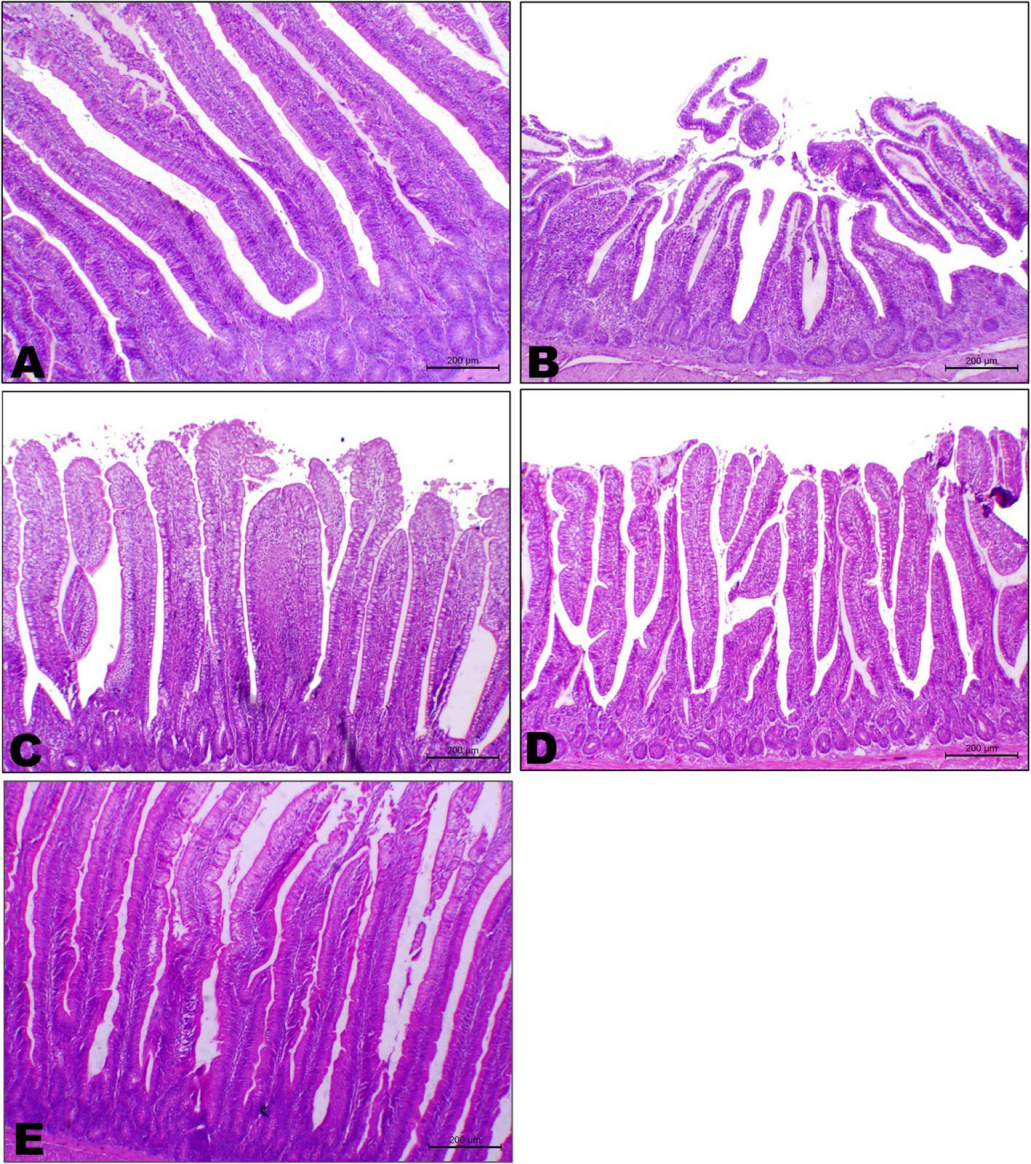
Effect of dietary Nano curcumin and Saccharomyces cell wall on photomicrograph of intestine (jejunum section) for broiler chicks fed on diet containing 0.25 mg aflatoxin B1 / kg diet at 1 to 35 day stained with hematoxylin and eosin (**H**&**E**) where (**A**) G1: showing normal histology of intestinal villi, (**B**) G2: showing mucosal necrosis of the intestinal villi with presence of necrotic core associated with blunting and villous atrophy, (**C**) G3: showing marked decrease of mucousal atrophy with an increase of villi length, (**D**) G4: showing mild degree of degenerative and desqumative changes within the intestinal villi with improvement of villi length, (**E**) G5: showing normal histology of intestinal villi with marked improvement of the thickness of mucosa, bar = 500 µm

On the other side, it was observed that the photomicrograph of the intestine (jejunum sections) of the control group (G1) showed normal histology of intestinal villi with normal pseudostratified epithelium with goblet cells (Fig. 2A). Conversely, the addition of the aflatoxin had an effect on intestinal tissue histopathology where a photomicrograph of the intestine (jejunum section) of the aflatoxin group (G2) showed mucosal necrosis of the intestinal villi with the presence of a necrotic core associated with blunting and with villous atrophy and focal basophilic regenerated epithelium (Fig. 2B). Meanwhile, the photomicrograph of the intestine (jejunum section) of the aflatoxin with *Saccharomyces* alone group (G3) showed a marked decrease in mucosal atrophy with an increase in villi length (Fig. 2C). On the other side, the photomicrograph of the intestine (jejunum section) of the aflatoxin with the Nano alone group (G4) showed a mild degree of degenerative and desquamative changes within the intestinal villi with an improvement in villi length (Fig. 2D). A photomicrograph of the intestine (jejunum section) of the aflatoxin with two treatments in combination group (G5) showed normal histology of intestinal villi with marked improvement in the thickness of intestinal villi and the structure of the lining mucosa (Fig. 2E).

Intestinal morphometrical data of intestinal sections of different treated groups showed a marked shortening of villi length in aflatoxicated birds, with a decrease in both crypt depth and villi length ratio when compared with their corresponding data of normal birds. Treatment of aflatoxicated birds with either *Saccharomyces* or nanocurcumin or *Saccharomyces* separately showed an increase in intestinal length and crypt depth in comparison with diseased birds. Interestingly, treatment of diseased birds with a combination of nanocurcumin and *Saccharomyces* demonstrated marked retrieval of intestinal parameters including villi length, crypt depth, and their ratio to the normal limits (Table 5).

**Table 5:** Effect of dietary Nano curcumin and *Saccharomyces* cell wall on intestinal morphometrical data of intestinal sections for broiler chicks fed on diet containing 0.25 mg aflatoxin B1 / kg diet at 1 to 35 day

Groups Items	**G1**	**G2**	**G3**	**G4**	**G5**
Villi length (µm)	1666.85 ± 71.21^c^	680.75 ± 34.82^a^	985.99 ± 32.90^b^	1153.26 ± 24.13^d^	1428.94 ± 19.97^d^
Villi width (µm)	119.02 ± 5.29 ^e^	246.43 ± 4.72 ^a^	164.74 ± 14.83^b^	128.27 ± 15.72^d^	97.23 ± 13.73^c^
Crypt depth (µm)	223.55 ± 8.82^b^	136.16 ± 13.69^a^	167.12 ± 7.75^c^	175.98 ± 5.30^e^	201.22 ± 6.94^d^
Goblet cell (No/mm2)	208.74 ± 4.24^b^	117.89 ± 8.82^c^	195.49 ± 7.57^b^	185.15 ± 3.40^a^	235.41 ± 4.96^a^
Crypt/Villi ratio	7.49 ± 0.49 ^a^	2.77 ± 0.19 ^b^	5.93 ± 0.29 ^a^	6.56 ± 0.11 ^a^	7.12 ± 0.19 ^a^

Values are means ± standard error

Mean values with different subscript letters (a-e) at the same row significantly differ at (*P* ≤ 0.05). G1 = group fed control diet without supplement; G2 = group fed control diet with aflatoxin; G3 = group fed control diet with aflatoxin and saccharomyces cell wall; G4 = group fed control diet with Nano curcumin; G5 = group fed control diet with saccharomyces cell wall and Nano curcumin

### Immunohistochemical study

**Fig. 3:**
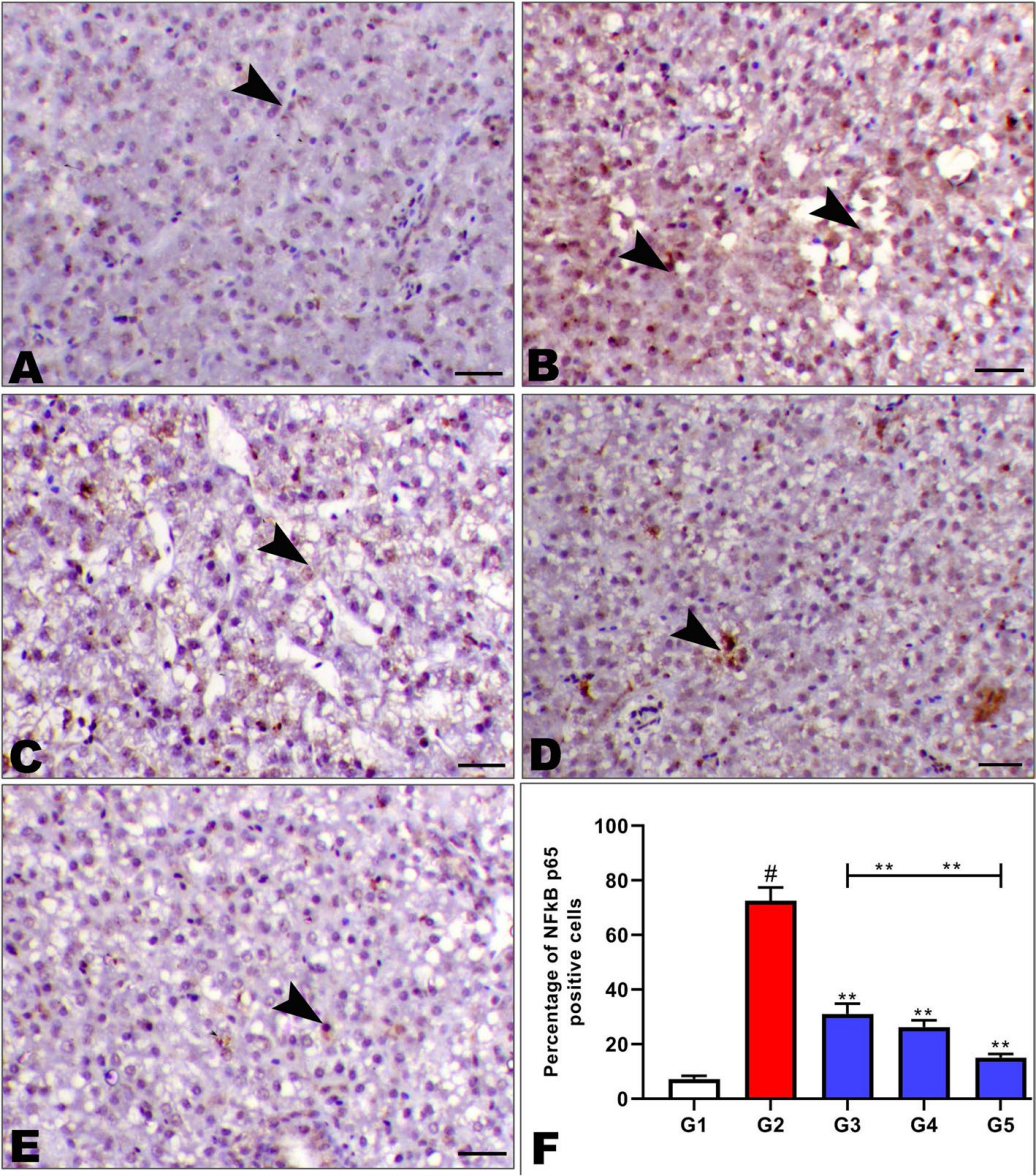
Effect of dietary Nano curcumin and Saccharomyces cell wall on immunostaining of NFkB P65 of the liver for broiler chicks fed on diet containing 0.25 mg aflatoxin B1 / kg diet at 1 to 35 day where (**A**) G1: showing mild immunostaining of NFkB P65 antibody within the hepatocytes (arrowheads), (**B**) G2: showing marked cytoplasmic and nuclear expression of NFkB P65 antibody within the hepatocytes (arrowheads), NFkB P65 IHC, (**C**) G3: showing decrease the expression of NFkB P65 antibody within the hepatocytes (arrowheads), (**D**) G4: showing decrease the expression of NFkB P65 antibody within the hepatocytes (arrowheads), NFkB P65 IHC, (**E**) G5: Liver of of aflatoxin + combination group showing marked decrease the NFkB P65 expression within the hepatocytes (arrowheads), (**F**) showing percentage of NFkB P65 positive cells in different groups, NFkB P65 IHC, X200, bar = 40 µm. B: nfkb p65 labelling index

An immunohistochemical study of the broiler liver is shown in Fig. 3, which was fed on an aflatoxin-free diet, aflatoxin diet, and aflatoxin diet with *Saccharomyces* cell wall or nanocurcumin or in combination.

It was observed that the broiler chick group that was fed an aflatoxin-free diet showed mild immunostaining of NFkB P65 antibody within the hepatocytes (Fig. 3A). Conversely, the addition of aflatoxin (G2) sharply upregulated the NFkB P65 in the liver tissue, where (Fig. 3B) showed marked cytoplasmic and nuclear expression of NFkB P65 antibody within the hepatocytes. Meanwhile, the expression of the NFkB P65 in the liver tissue of broiler chicks was fed with aflatoxin either with *Saccharomyces* cell wall (G3) or with nano (G4) showed a decrease in the expression of the NFkB P65 antibody within the hepatocytes (Fig. 3C and D). On the other side, the expression of the NFkB P65 in the liver tissue of the aflatoxin with two treatments in combination group (G5) showed a marked decrease in the upregulation of the NFkB P65 antibody within the hepatocytes (Fig. 3E). The percentage of NFkB P65 positive cells was markedly increased in G2 and decreased in G3, G4, and G5 (Fig. 3F).

### Expression of *CYP1A1* and *Nrf2* genes within the liver tissue of broilers

**Fig. 4:**
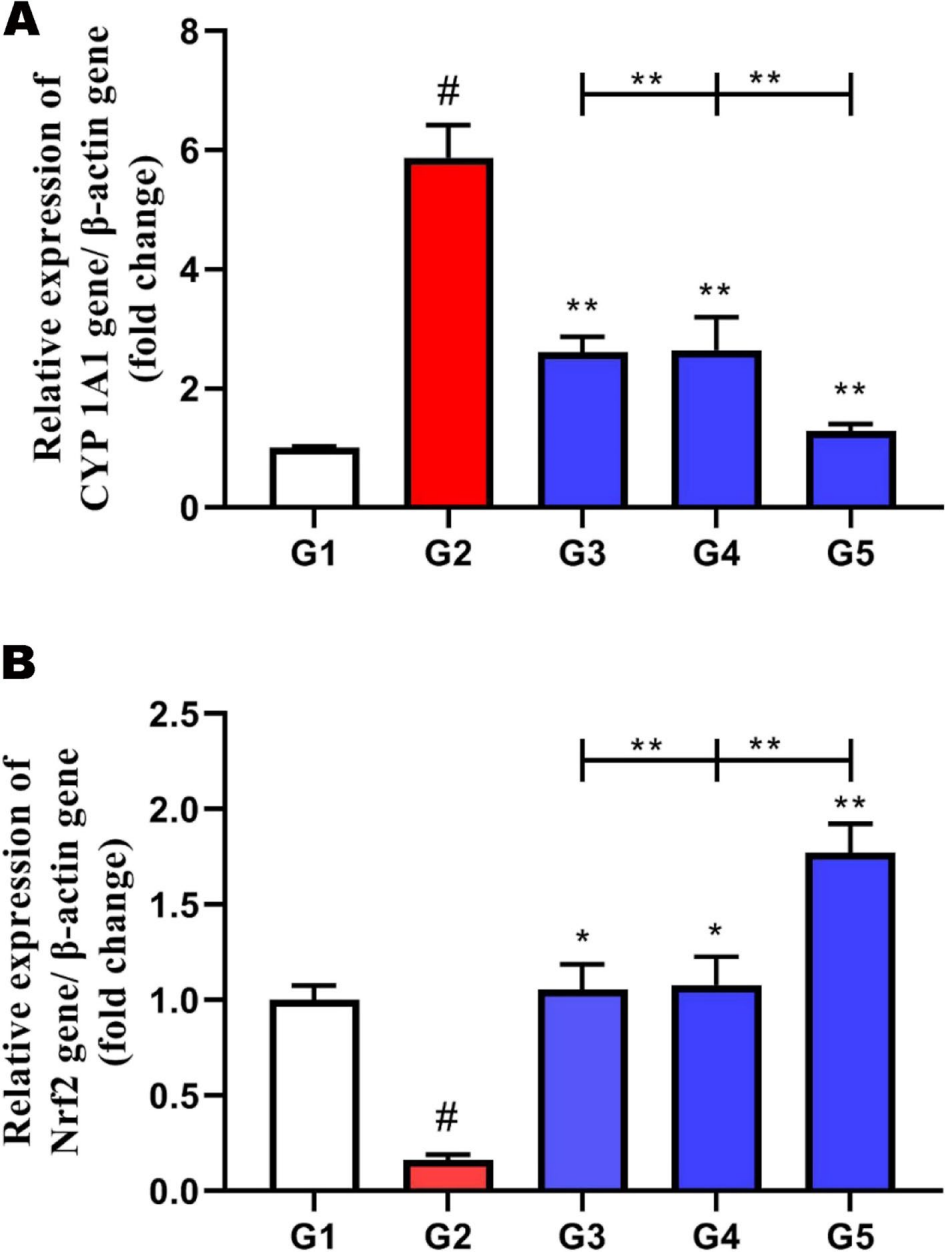
Effect of dietary Nano curcumin and Saccharomyces cell wall on some genes expression in the liver for broiler chicks fed on diet containing 0.25 mg aflatoxin B1 / kg diet at 1 to 35 day where (**A**) Expression of *CYP 1A1* gene within the liver tissue, (**B**) Expression of *Nrf2* gene within the liver tissue. Data are expressed as mean ± SD, and superscript samples # and ** indicate significance in comparison with the control group

The responses of some genes related to carcinogens and antioxidants as *CYP1A1* (Cytochrome P450 Family 1 Subfamily A Member 1) and *Nrf2* (Nuclear Factor Erythroid 2-related Factor 2), respectively, are illustrated in Fig. 4. The expression of the *CYP1A1* gene was markedly increased in the aflatoxin group (G2) in comparison with the control (G1) group (*P* > 0.005). There was a marked decrease in this gene with supplementation of *Saccharomyces* cell wall (G3) or nano (G4) or two treatments in combination (G5) relevant to the aflatoxin group (*P* > 0.005). Liver tissues of aflatoxin (G2) animals revealed a marked decrease of *Nrf2* compared to the normal (G1) group (*P* > 0.005), which increased to the normal limits of control groups and significantly increased in aflatoxicated-birds supplemented with *Saccharomyces* cell wall (G3) or nano (G4) or two treatments in combination (G5) than the aflatoxin group (*P* > 0.05).

## Aflatoxin residue within the liver and muscle tissue of broilers

Aflatoxin residues in the liver and muscle tissue at the end of the experiment in all groups and two weeks after the end of the experiment in the liver tissue of the G2 (aflatoxin group) are illustrated in Table 6. The aflatoxin residue at the end of the experiment in both liver and muscle tissue was markedly increased in the aflatoxin group (G2) in comparison with the other groups (*P* ≤ 0.05). There was a marked decrease of this residue with the supplementation of *Saccharomyces* cell wall relevant to the aflatoxin group (*P* ≤ 0.005). Liver tissue of aflatoxin animals at 2 weeks after the end of the experiment revealed a marked decrease in aflatoxin residue when compared with the result of G2 at the end of the experiment (*P* ≤ 0.05).

**Table 6:** Effect of dietary Nano curcumin and *Saccharomyces* cell wall on aflatoxin residues values for broiler chicks fed on diet containing 0.25 mg aflatoxin B1 / kg diet at 1 to 35 day

Groups Items	**G1**	**G2**	**G3**	**G4**	**G5**
Residue in the liver tissue at the end of the experiment (µg/kg)	0.0 ± 0.0 ^c^	13.13 ± 0.32^aX^	2.90 ± 0.30 ^b^	0.0 ± 0.0 ^c^	0.0 ± 0.0 ^c^
Residue in the liver tissue at 2 weeks after the end of the experiment (µg/kg)	-	0.80 ± 0.20^Y^	-	-	-
Residue in the muscle tissue at the end of the experiment (µg/kg)	0.0 ± 0.0 ^c^	5.77 ± 0.25 ^a^	2.17 ± 0.21^b^	0.0 ± 0.0 ^c^	0.0 ± 0.0 ^c^

Values are means ± standard error

Mean values with different subscript letters at the same row (a-c) and same column (X–Y) significantly differ at (*P* ≤ 0.05). G1 = group fed control diet without supplement; G2 = group fed control diet with aflatoxin; G3 = group fed control diet with aflatoxin and saccharomyces cell wall; G4 = group fed control diet with Nano curcumin; G5 = group fed control diet with saccharomyces cell wall and Nano curcumin

## Discussion

### Clinical signs and postmortem lesions of the aflatoxicated broilers

The clinical symptoms of the aflatoxicated broilers in the present trial are compatible with the results of Salem et al. [22], who reported that the aflatoxicated birds show retarded growth, leg paralysis, and lameness, as well as wing paralysis. These results may be attributed to the effect of aflatoxin on the nervous system as the sciatic nerve, leading to nerve injury [23]. On the other side, there is whitish diarrhea that indicates infection because of immune deficiency that leads to pathogenic microbes flushing out [24]. Tessari et al. [23] reported that aflatoxin decreases protein because of liver injury that leads to immune suppression. Regarding the results of mortality and lesion of euthanized birds, there was no mortality in the aflatoxicated broilers with enlarged liver and kidney accompanied with generalized congestion in the muscle tissue. These results were confirmed by the results of Salem et al. [22]. However, these adverse effects of aflatoxin on clinical signs and internal organs were alleviated by the addition of *Saccharomyces* cell wall/Nanocurcumin alone/in combination to the aflatoxin-contaminated diet. These results are confirmed by the results of Karaman et al. [25], who found that aflatoxicated chick livers and kidneys were usually enlarged with a pale yellow-red colour and that these adverse effects on the internal organs decreased with yeast glucomannan supplementation. Furthermore, curcumin reduces the clinical signs and postmortem lesions of aflatoxicated broilers, according to Raja et al. [26].

### Growth performance

The obtained results regarding growth performance showed a significant reduction in the mean values of final body weight, BWG, and feed intake in the aflatoxicated group (G2). The impairment of metabolizing capability may be the cause of AFB1's negative influence on growth parameters [27]. These findings are in harmony with previous studies reporting the negative effects of aflatoxin on growth performance [5, 21, 28]. Moreover, Mahrose et al. [29] reported the deleterious effects of aflatoxin on the growth performance of Japanese quail. These results are incompatible with the results of dos Santos et al. [30], who reported that AFB1 has no deleterious effects on the broilers’ performance after feeding for 21 days. Moreover, Mahmood et al. [31] reported that there was no difference in the growth performance of broilers that were fed an aflatoxin-free diet and an aflatoxicated one. This difference may be attributed to the period of the aflatoxin exposure (experimental period), where in our study the experimental period was 35 days whereas in the dos Santos et al. trial it was 21 days. In addition to, species and sex Mahrose et al. [29]. On the other side, the addition of *Saccharomyces* cell wall/Nanocurcumin alone to the aflatoxin-contaminated diet ameliorated the toxic effects of aflatoxin B1 on growth performance in broilers fed on for 35 days. These results are assured by the results of Gowda et al. [32] and Liu et al. [33] for *Saccharomyces* cell wall or Nanocurcumin alone, respectively. The positive effects of *Saccharomyces* cell wall may be attributed to its action as a biodegradable agent with toxins that function as antioxidants by stimulating enzyme synthesis and promoting weight gain by improving vitamin and mineral absorption and protein metabolism [34]. Supplementing with biodegradable agents also has an effect on the digestive system, promoting the production of digestive enzymes that are important for better digestion and, as a result, weight gain [35, 36]. In contrast, Nemati et al. [37] reported that glucomannan administration does not prevent or decrease the aflatoxin deleterious effects on broilers’ growth parameters. Moreover, Santin et al. [38] found that *Saccharomyces* cell wall does not ameliorate the negative effect of AFB1 on weight gain but improves the FCR of the broilers. In fact, to our knowledge, there are no publications on the combination of both nanocurcumin and *Saccharomyces* cell walls on aflatoxicated birds.

### Carcass traits

It is normal for the growth results to affect the results of the carcass. The result of the present study showed a decrease in the dressing percentage of the broilers that fed on the aflatoxin-contaminated diet for 35 days (G2) compared with the other groups. These results are in line with the findings of Pasha et al. [39] and dos Santos et al. [30]. On the other side, the addition of *Saccharomyces* cell wall alone ameliorated the toxic effects of aflatoxin B1 on carcass traits in broilers fed on for 35 days. These findings are compatible with the results of dos Santos et al. [30], who reported that the *Saccharomyces* cell wall improves the carcass yield and characteristics of aflatoxicated broilers. Moreover, the significant increase of the breast muscle in the group that received *Saccharomyces* cell wall and Nanocurcumin in combination to the aflatoxin-contaminated diet (G5) may be attributable to the favorable effect of nanocurcumin in elevating the crude protein content of broiler breast meat [40] and the protective effects of the functional ingredients in YCW (beta-glucan and mannan oligosaccharides) [41]. As well, the relative weight of the liver in the aflatoxicated group (G2) showed a significant increase when compared with the other groups. These findings are similar to those of Dhanapal et al. [6], who reported that there was a significant increase in relative liver weight in broilers fed on a basal diet contaminated with 1 ppm aflatoxin from day 14 to 28 post-treatment. In the current study, the addition of *Saccharomyces* cell wall alone to the aflatoxin-contaminated diet significantly increased thymus relative weight in broilers fed on for 35 days as reported by Mendieta et al. [42], but regarding the relative weight of bursa, the findings are in contrast with the present study as bursa is insignificantly increased in the aflatoxin treated group (G2).

### Hematological parameters

The hematological toxic effects of AF are a well-investigated and well-known subject. These toxic effects were also clearly observed in the present study. There was a significant decrease in RBCs count, HCT, WBCs count, and LYM in the G2 when compared with the other groups. These results are compatible with those of Basmacioglu et al. [43], who reported the same result when AF was incorporated into the diet of broiler chicks. Aflatoxin treatment was reported to induce a significant increase in HET, ESI, and MON counts [8], and this is what our study proves, HET, ESI, and MON counts showed a significant increase in G2 compared with the other groups. These results may be attributed to the inflammatory response that is caused by aflatoxin and leads to heterophilia [44]. Moreover, aflatoxin leads to immune deficiency [45] as a result of protein defiance. Thus, the birds are exposed to infection, especially, parasitic infection, as indicated by whitish diarrhea in this study. This infection leads to eosinophilia and monocytosis. On the other hand, the beneficial effect of the addition of *Saccharomyces* cell wall alone to the aflatoxin-contaminated diet on the hematology in broilers fed on for 35 days was reported in a previous study [42]. Moreover, Rahmani et al. [46] reported that nanocurcumin improves the hematological parameters of broiler chicks under cold stress. This improvement may be attributed to the antioxidant effect of nanocurcumin [47].

### Biochemical parameters

The target organ for aflatoxin detoxification is the liver, where reactive 8,9-epoxide is formed through the activation of aflatoxin. This active form can bind to proteins and DNA. Thus, the measurement of the liver enzyme indicates liver injury as a result of aflatoxin [48]. Thus, in the present trial, there was a significant increase in the ALT, AST, and ALP activities in the aflatoxin group, as reported by previous studies [1, 22, 49, 50], and this is evidence of the existence of liver injury due to toxicity. Conversely, these outcomes are different from this previous study [51], which explained that AFB1 does not alter serum ALT and AST activities. Similar to many studies [52–56], the addition of Nanocurcumin alone to the aflatoxin-contaminated diet alleviated the toxic effects of aflatoxin B1 on the liver enzymes in broilers fed on for 35 days. Where the levels of the liver enzymes decreased by preserving the structural integrity of the cell membrane, as shown in G4 compared to the other groups. In addition, ALT, AST, and ALP were significantly reduced by YCW. This result is compatible with many studies [57, 58], which explain the effect of yeast cell wall extract on liver enzyme activities. The significant decrease of AST and ALP in the G4 may be due to the free radical scavenging activity of nanocurcumin [56]. Aflatoxins are the source of a wide range of metabolic losses, and changes in blood biochemicals are a sign of liver damage and metabolic route disruption [59]. Abd El-Ghany et al. [60], Gholami-Ahangaran et al. [61], and Naseem et al. [62] observed a significant decrease in total protein, albumin, and globulin in the aflatoxicated group compared to the group that was fed on a basal diet only. This is indicative of the toxic effect of aflatoxin B1 on the liver and kidneys and is an indicator of diminished protein synthesis [60]. As shown in our study, the addition of *Saccharomyces* cell wall/Nanocurcumin alone/in combination to the aflatoxin-contaminated diet alleviated the toxic effects of aflatoxin B1 on the hepatic function in broilers fed on for 35 days, where the serum total protein, albumin, and globulin concentrations were increased. These results are compatible with previous studies [63–65]. On the other hand, Li et al. [66] explained that YCW incorporation did not affect the same biochemical indices, including total protein, albumin, and globulin. Moreover, Emadi et al. [14] reported the hepatoprotective properties of curcumin in Japanese quail. Regarding results of kidney function, many previous studies [67–70] showed that the inclusion of aflatoxin in the diet led to a significant increase in kidney function, including urea, creatinine, and uric acid. In contrast, our results showed that the concentrations of urea and uric acids were insignificantly increased in G2 when compared with the other groups. On the other side, the creatinine concentration was significantly increased in G2 when compared with G1 but non-significant when compared with the other groups. However, total blood protein is very important for the transportation of vitamins, hormones, enzymes, and electrolytes. Also, albumin is represented in this total protein, where it makes up a significant portion of it. On the other hand, the indictors of protein metabolism are creatinine and uric acid, which form new tissues and function of the kidney [29]. These facts confirmed our results where aflatoxin affected the liver, resulting in decreased total protein and increased creatinine and consequently, affecting growth performance.

### Histopathological finding

Aflatoxin has a direct effect on the liver, which is a primary target organ. Aflatoxin has been shown in numerous studies [51, 71, 72] to be toxic to hepatic tissues. The addition of Nanocurcumin alone to the aflatoxin-contaminated diet ameliorated the toxic effects of aflatoxin B1 on the liver tissue histopathology. These positive effects come in contrast to Sayrafi et al. [16], who investigated the protective effects of nanocurcumin on the liver toxicity induced by salinomycin in broiler chickens. Moreover, the addition of *Saccharomyces* cell wall alone to the aflatoxin-contaminated diet alleviated the toxic effects of aflatoxin B1 on the liver tissue photomicrograph, which showed the same results as Yalçin et al. [20]. On the other side, the addition of *Saccharomyces* cell wall and Nanocurcumin in combination to the aflatoxin-contaminated diet showed the normal photomicrograph of the liver. These results may be due to the bio adsorbing feature of the functional ingredients in YCW (beta-glucan and mannan oligosaccharides ) as reported by Yiannikouris et al. [41], and the detoxification effect of curcumin nanoparticles as reported by Yu & Huang [73].

Aflatoxin has been shown to destroy the normal morphology of the intestine [74]. However, there are a few reports on the effect of AFB1 on jejunal apoptosis [75]. Our results on the effect of aflatoxin on the jejunum section are compatible with Peng et al. [75], Jahanian et al. [5], and Wang et al. [76]. Yeast and yeast components, especially *Saccharomyces cerevisiae* cell walls, are recent approaches to eliminate side effects of AFB1 [77]. Moreover, Reisinger et al. [78] investigated the effects of yeast derivatives (yeast cell wall fragments and yeast extract derived from *Saccharomyces cerevisiae*) on the jejunum of broiler chickens. They concluded that birds fed 0.1% and 0.2% yeast derivatives have a significantly higher density of goblet cells (number per 10 µm of villi length) than the control group.

Previous studies have shown that curcumin detoxification mechanisms, including inhibition of AFB1 phase I enzyme-mediated biotransformation and phase II enzyme activity up-regulation, can control the carcinogenic effects induced by AFB1 and the toxic effects attributed to the positive regulation of cells [79]. Therefore, the present study found that the photomicrograph of the intestine (jejunum section) of the aflatoxin with Nano alone (G4) group showed a mild degree of degenerative and desquamative changes within the intestinal villi with improvement of villi length compared to the aflatoxin group, similar to this study by Rahmani et al. [46]. The last author found that villus height (VH), villus height/crypt depth ratio, and the villus surface area (VS) were higher in birds fed curcumin / nanocurcumin supplements than in those fed control food. Furthermore, our study showed that a photomicrograph of the jejunum section of aflatoxin with two treatments in combination (G5) group showed normal histology of intestinal villi with a marked improvement in the thickness of the mucosa. This is due to the bio adsorbing feature of the functional ingredients in YCW (beta-glucan and mannan oligosaccharides) [80] and the detoxification effect of curcumin nanoparticles [73].

### Immunohistochemical study

The nuclear factor-kappa B (NF-KB) transcription factor is made up of homo- and heterodimeric complexes formed by the Rel family of proteins. There are five members of the Rel/ NF- KB proteins in vertebral cells, including p65 (Rel A), p50/p105, p52/100, c-Rel, and Rel B. The most common NF- KB is a heterodimer composed of p65 and p50 [81]. Ma et al. [82] reported that AFB1 treatment increased the protein levels of NF-KB p65. Moreover, when Fadl et al. [65] studied the effects of dietary Mannan-oligosaccharide and β-Glucan (yeast cell wall extract) supplementation on hepatic transcripts of NF-KB of broilers challenged with *E. coli* O78, they found that the gene expression of NF KB was reduced, and this indicates the protective effects and anti-inflammatory properties of YCW extract in broilers. This result is similar to the current result in spite of the fact that the current result obtained the expression of the NFkB P65 itself in the liver tissue of AF affected broilers. Moreover, the present results showed that nanocurcumin decreases the expression of NFkB P65 antibodies within the hepatocytes. This result is consistent with that obtained by Chang et al. [83] in the brain cells of rats. On the other side, the expression of the NFkB P65 in the liver tissue of the aflatoxin with two treatments in combination (G5) group was markedly decreased, which may be due to the anti-inflammatory and protective effects of the functional ingredients in YCW (beta-glucan and mannan oligosaccharides ) [41], and the detoxification effect of curcumin nanoparticles [73].

### Expression of *CYP1A1* and *Nrf2* genes within the liver tissue of broilers

Similar to the current result regarding the expression of the *CYP1A1* gene in the liver tissue of broiler chickens, Zhang et al. [1] found that there is a marked increase in the level of *CYP1A1* in the aflatoxin group compared with the control. As well, Zhao et al. [51] reported that AFB1 decreases *Nrf2* protein levels in broiler livers. El-Gendy et al. [84] reported that *Nrf2* protein levels significantly increased in aflatoxicated-birds supplemented with *Saccharomyces* cell wall. In addition, Zaki et al. [85] concluded that there was a decrease in *Nrf2* protein levels with the use of curcumin and nanocurcumin compared to that of deltamethrin-treated rats, and this is what our study proves.

### Aflatoxin residue within the liver and muscle tissue of broilers

Residual AFB1 in the liver not only affects the performance and health of broiler chickens but also adversely affects the health of consumers of broiler products due to aflatoxin accumulation in the edible parts of poultry. Therefore, it is necessary to control the quality of poultry products and analyze aflatoxin residues in various poultry tissues, taking into account public health and safety [22]. The result in the present study of the aflatoxin residue in G2 is in line with the findings of Hussain et al. [49]. *Saccharomyces cerevisiae* cells are encased in a cell wall that is formed from an extracellular matrix made up of a layered meshwork of -glucans, chitin, and mannoproteins [86]. Mycotoxins' harmful effects have been found to be prevented by glucan-based binders that adhere to them during digestion and inhibit their absorption from the gastrointestinal system [87]. Thus, the aflatoxin residue was decreased in the G3 as a result of the binding of aflatoxin with the glucan of the *Saccharomyces cerevisiae* cell wall [88]. These results are confirmed by the results of Yiannikouris et al. [41], who reported the adsorbent effect of *Saccharomyces crevice* cell wall against aflatoxin in rats. Also, Oğuz et al. [89] reported the adsorbent effect of *Saccharomyces crevice*. On the other hand, Limaye et al. [90] reported that curcumin inhibits cytochrome P450 isoenzymes, notably the CYP2A6 isoform, lowering the generation of aflatoxin metabolites such as AFB1-8, 9-epoxide, and other toxic metabolites. Where the active form of aflatoxin (AFB1-8, 9-epoxide) can bind to proteins and DNA [48]. However, nanocurcumin is used to improve the bioavailability of curcumin [64]. Thus, the effect of Nano curcumin will surely be stronger than curcumin itself. In addition, in the liver, AFB1 is linked to the levels of antioxidant enzymes where lower levels of these enzymes, higher levels of MDA, 8-hydroxydeoxyguanosine, and AFBO-DNA and curcumin increase the levels of antioxidant enzymes [91].

## Conclusion

This trial introduces the new importance of the addition of *Saccharomyces* cell wall (1kg YCW/ton diet)/Nanocurcumin (400 mg NC/kg diet) alone/in combination to the aflatoxin-contaminated diet (0.25 mg AFB1/kg diet). It considerably ameliorated the toxic effects of aflatoxin B1 on growth performance, blood and serum parameters, carcass traits, aflatoxin residue in organs, intestinal villi, and the expression of some genes in the liver tissue of broilers fed on for 35 days. Thus, inconclusion, dietary supplementation of *Saccharomyces* cell wall/Nanocurcumin alone/in combination may reduce the negative effects of many mycotoxins besides aflatoxin B1. However, the result of the aflatoxin residue in the liver and muscle tissue is of great public health importance. The best results were obtained by the addition of Nanocurcumin alone/in combination with *Saccharomyces.* In addition, the aflatoxin residue in the liver tissue decreased in the aflatoxicated group that was fed an aflatoxin-free diet for two weeks after the end of the experiment, so it is possible to predict the withdrawal time of aflatoxin from broiler tissue.

## Methods

### Chemicals and treatments

Aspartate aminotransferase (AST), alanine aminotransferase (ALT), alkaline phosphatase (ALP), total protein, albumin, creatinine, urea, and uric acids were purchased from Biodiagnostics Company (Dokki, Giza, Egypt). The RNA extraction Kit was purchased from Introbio Company, Cat.No. 17063. Reverse transcriptase Polymerase chain reaction (RT-PCR) sensiFAST™ cDNA synthesis kit (Bioline, United Kingdom). All used primers are commercially based. Aflatoxin from the Mycology and Mycotoxins Department, Animal Health Research Institute (ARC), Dokki, Egypt. Curcumin Nanoparticles (NT-Cur-NPs) were purchased from Nanotech Egypt for photo- electronics at the city of 6 October, Al Giza. *Saccharomyces cerevisiae* cell wall extract (100%, Beta-glucan: 20% minimum &Manan Oligosaccharides: 20% minimum.) was obtained from IBEX Group for animal nutrition and health products and produced by Shandong Bio Sunkeen Co., Ltd (a state-owned high-tech enterprise that integrates R&D, production and sales with rich experience of yeast and nature yeast products.

### Experimental birds, feeding, and design

One hundred and fifty one-day-old Cobb chicks with an average initial body weight of 55.6 g were obtained from a local hatchery. The birds were individually weighed, randomly allocated into 5 groups (30 chicks per group), and each group was subdivided into three replicates (10 chicks per each). The basal diets were formulated in Table 7 according to NRC [92]. The experimental feeding was designed as follows:

- Group 1: Basal diet
- Group 2: Basal diet with 0.25 mg/kg AFB1 [22]
- Group 3: Basal diet with 0.25 mg /kg AFB1 + Lancell YCW 1 kg/ton according to Shandong Bio Sunkeen Co., Ltd & IBEX group
- Group 4: Basal diet with 0.25 mg /kg AFB1 + NC 400 mg/kg [46]
- Group 5: Basal diet with 0.25 mg /kg AFB1 + YCW 1 kg/ton + NC 200 mg/kg [46].

**Table 7:** Ingredients and calculated chemical composition of the used basal diets

Ingredients	Ingredients%		Chemical composition		
	Starter	Grower and finisher	Items	Starter	Grower and finisher
Yellow Corn	54.5	60	ME (Kcal/Kg)	3201.5	3221
Soybean (44%)	27.27	20	Crud protein%	23	20.08
Corn gluten meal (62%)	10	8.73	Calcium %	1	0.9
Wheat bran	0	3.5			
Sunflower oil	4	4	Available phosphorus%	0.45	0.35
Dicalcium phosphate	1.78	1.3	Lysine%	1.1	1
Limestone	1.3	1.4	Meth. + Cyst%	0.9	0.72
Lysine	0.13	0			
DL-Methionine	0.1	0.2			
Common salt	0.4	0.4			
Choline chloride (60%)	0.22	0.17			
Premix^a^	0.3	0.3			

^a^The used premix (*Multivita Co.*) composed of vitamin A 12,000,000 IU, vitamin D_3_ 2,200,000 IU, vitamin E 10,000 mg, vitamin K_3_ 2000 mg, vitamin B_1_ 1000 mg, vitamin B_2_ 5000 mg, vitamin B_6_ 1500 mg, vitamin B_12_ 10 mg, Niacin 30,000 mg, Biotin 50 mg, Folic acid 1000 mg, Pantothenic acid 10,000 mg, Iron 30,000 mg, Manganese 60,000 mg, Copper 4000 mg, Zinc 50,000 mg, Iodine 1000 mg, Cobalt 100 mg, Selenium 100 mg, calcium carbonate (CaCO_3_) carrier to 3000 g

### Housing and management

The birds were reared (the trial period was 35 days) in a clean, well-ventilated room, which was fumigated and disinfected with formaldehyde gas (obtained by mixing 40% formalin and potassium permanganate powder). The proper temperature of the room was obtained by using gas heaters and electric lamps (200 watts) in each partition, which were light and dark for 23/1 hrs./day. The floor of the room was divided into 15 divisions. Each compartment was covered with fresh, clean straw, forming a deep litter 4 centimeters deep. Each compartment was provided with an appropriate feeder and waterer.

Prophylaxis against the most common infectious diseases is carried out using Spectrama Vet (1 ml/2 liters), Colistin sulfate (1g/4 liters) for *Salmonellosis & E. coli* infections for the first three days, and Coxistac (0.1%) of the diet) for coccidiosis. Chicks were vaccinated against Newcastle disease, IB, and Gumboro. After vaccination, chicks were given vitamin AD3E (1ml/L drinking water) to improve their vitality. The body weights (BW) of the chicks and their feed intake were weighed at weekly intervals to calculate the other growth performance parameters.

### Blood and serum samples

Birds were anaesthetized on day 35 by an intraperitoneal injection of sodium pentobarbital (50 mg/kg) to minimize suffering during blood sample collection. Blood samples (3ml/bird) were taken from the vena brachialis under the wings of 5 birds in each group using 2 different capillary tubes for each one, an EDTA tube for haematological evaluation [93] and another without anticoagulant for serum separation. Blood samples were clotted at room temperature. Serum separation was performed by centrifuging the coagulated blood at 3000 rpm for 15 minutes. Serum was collected and stored at -20°C for determination of urea, creatinine, albumin, total protein, uric acid, and activity of AST, ALT, and ALP using commercial kits, spectrophotometrically.

### Tissue samples

The anaesthetized broilers were sacrificed through cervical dislocation. After taking carcass and organ weight**,** sections of the liver, kidney, and intestine were immediately fixed in 10% formalin for histopathological examination. Liver samples were also taken and kept at -80°C for (RT- PCR) to determine mRNA expression of cyp450 1A1 and Nrf2. To detect aflatoxin residue in the liver and muscle tissue, liver and muscle samples were collected from 5 birds of each group. also on day 50, liver and muscle samples were collected from the aflatoxin-fed group (G2) only and were stored in a deep freezer until use.

At the end, CO2 suffocation was used to suffocate the remaining living birds in thick sacks. All of the birds (125), including dead and cervical dislocated birds, as well as samples and bedding material remnants, were buried in a hygienically regulated, well-constructed burial hole.

### Carcass traits

Dressing percentage: five birds from each group were eviscerated, weighed without feathers and heads and the dressing percentage was calculated according to the following formula:

### Dressing % = dressed carcass weight/Live weight X 100

Relative organ weights: Heart, gizzard, proventriculus, liver, spleen, thymus, bursa, and breast and leg muscles were weighed and their relative weights to body weight were calculated.

### Histopathological examination

Following necropsy, liver and intestinal sections were obtained and promptly fixed in 10% buffered formalin before being processed for histological examination using conventional paraffin sections. Bancroft & Gamble [94] recommended cutting 5 m thick sections and staining them with hematoxylin and eosin (H&E).

### QRT-PCR analysis

Reverse transcription-quantitative polymerase chain reaction (RT-qPCR) was used to analyze the gene expressions. Total RNA from the liver was extracted using the TRIzol® reagent (Invitrogen, USA). Then, reverse- transcription of the extracted RNA was done to create cDNA. Table 8 shows the employed primer sequences for the following; β-actin, *CYP1A1*, and *Nrf2* genes. RT-qPCR reactions were completed by Power SYBR® Green PCR Master Mix (Applied Biosystems, USA). Reactions were performed by the 7500 Real-Time PCR System (Applied Biosystems, USA). Thermal cycles were performed at 95°C for 4 minutes, 40 cycles of 10 seconds each at 95°C, 30 seconds at 60°C, and finally 10 seconds at 72°C. Data were presented as relative fold changes compared to the control’s gene expressions.

**Table 8:** Primers used for qRT-PCR analysis

Gene	Gene bank No	Forward primers Sequence (5ʹ-3ʹ)	Reverse primers Sequence (5ʹ-3ʹ)	Product size (bp)
β-actin	L08165	ATGGCTCCGGTATGTG C AA	TGTCTTTCTGGCCCATACCAA	178
*CYP1A1*	X99454.1	CACTTTCTGCCTGCTCCTG	GGTCCTTCCTCAGCTCCAG	125
*Nrf2*	NM_001030756.1	CTGCTAGTGGATGGCGAGAC	CTCCGAGTTCTCCCCGAAAG	132

Method for detection of aflatoxin residue in the liver and muscle tissue

The liver and muscle samples (5 samples/group) were taken after 35 days from the beginning of the experiment and after the withdrawal of AFB1 from the diet of the aflatoxicated group (G2), (after the end of the experiment by 2 weeks). The samples were stored in a deep freezer until used. According to Schuller and Van Egmond [95], the AFB1 residue was estimated in the liver and muscle samples by thin-layer chromatography (TLC).

### Statistical analysis

To assess significant differences, the obtained data were statistically analysed using one-way analysis of variance with SSPS. A value of *P* < 0. 05 was considered to be significant.

## Supplementary Information

**Additional file 1.**

**Additional file 2.**

## Data Availability

The datasets supporting the conclusions of this article are included within the article (and its additional files 1 and 2).
