# Supplementary material for: Ameliorative effect of nanocurcumin and Saccharomyces cell wall alone and in combination against aflatoxicosis in broilers
Source: BMC Vet Res. 2022 May 14;18:178. doi: 10.1186/s12917-022-03256-x (PMC9107200; doi:10.1186/s12917-022-03256-x)
Supplement: Supplementary file 1 — Additional file 1. [file 12917_2022_3256_MOESM1_ESM.docx]

**Immunohistochemical**

1. Neutral buffer formalin.
2. Dilution anti-NF-ĸB P65, Santa cruz, Cat# (F-6): sc-8008, 1:100 dilution).
3. PBS.
4. A goat anti-rabbit secondary antibody (cat. no. K4003, EnVision+™ System Horseradish Peroxidase Labelled Polymer; Dako).
5. DAB kit (3,3’-Diaminobenzidine).
6. Mayer's haematoxylin.

**Haematological material and devices**

- a slide and a rectangular coverglass (24 mm x 50 mm)
- Wright's stain
- Drabkin ‘s reagent
- microhematocrit tube
- calorimeter
- methyl violet 2B diluent
- Neubauerruled hemacytometer
- WBCs diluting pipette
- RBCs diluting pipette
- Diluting solution (Natt and Herrick)

**Histopathological material**

- Tissue specimens from liver, kidney, and intestine
- Formaldehyde 10%.
- Liquid nitrogen tank.
- Alcohol (100, 90, 80, 70 %), Nasr Co, Giza, Egypt.
- Paraffin wax.
- Microtome device for preparation of histopathological sections (Leica microtome, Germany).
- hematoxylin and eosin (H&E) stain
- Light microscope (Leica microscope supplied with DFC camera).

**Production of the aflatoxin.**

*Aspergillus flavus* microbes (toxigenic strain with gene bank accession number: KP137700) were isolated from the broiler’s diets and used for the production of aflatoxin B1 (AFB1). The production of AFB1 was performed at the Mycology Department, Animal Health Research Institute, Giza, Egypt. The Czapek yeast extracts were used for the subculture and growth of toxigenic strain for a week. According to **Shotwell et al. [1]**, Thin Layer Chromatography (TLC) was used for screening of toxigenic strain to produce AFs. The emergence of blue fluorescence on the plate and a comparison of the spot's Retention Factor (RF) value to that of a known standard were used to validate the presence of different AFs qualitatively. The gross and TLC examinations verified that a commercial crushed yellow corn was completely free of fungus or mycotoxins contaminations. The commercial crushed yellow corn was autoclaved at 121^O^C for fifteen min in the conical flasks for 3 successive days. After that, the autoclaved corn was treated with 10 ml of the spore suspension of the toxigenic strain (106 spores/ml). Then the treated corn was fermented by incubation at 28–30 °C for twenty-one days. After 21 days, the fungus in the incubated corn was killed in a 50 °C oven for 3-4 days. After that, the grinder was used for powdering the crushed corn. Then 25 g from the powdered corn as a representative sample was used to calculate AFB1 **[2]**. Then the contaminated corn was incorporated into the formulated broilers basal diet to provide the desired dose of 0.25 mg of AFB1/kg diet.

**Biochemical analysis**

- - - 1. **Serum albumin**

according to **Doumas et al. [3]**, Serum albumin was measured calorimetrically following the instruction defined in the pamphlet

**principle**

Formation of an albumin/bromcresol – green complex at pH 3.8 and photometric measurement of the absorbnance

**Reagents**

| 1- | Standard Albumin | 4 g/dl |
| --- | --- | --- |
| 2- | Color Reagent | 50 mmol/l |
|  | Citrate buffer, pH 3.8 |  |
|  | Bromcresol green | 0.12 mmol/l |
|  | Detergent |  |
|  | Preservative |  |

**Procedure**

|  | Blank (ml) | Standard (ml) | Sample(ml) |
| --- | --- | --- | --- |
| Standard | - | 0.01 | - |
| Sample | - | - | 0.01 |
| Reagent 2 | 2.0 | 2.0 | 2.0 |

Mix well, then measure after 5 min. the absorbances of sample (Asample) and the standard (Astandard) against reagent blank at 630 nm (620 – 640 nm).

**Calculations**

The serum albumin concentration is calculated by following formula

$$Serum albumin=\frac{A sample}{A standard} X 4 = g/dl$$

- - - 1. **Total protein**

According to **Gornall et al. [4]** Serum albumin was measured calorimetrically following the instruction defined in the pamphlet

**Principle:**

In the presence of an alkaline cupric sulfate, the protein produces a violet color, the intensity of which is proportional to their concentration.

**Reagents**

| 1**-** | Standard Albumin |  | 5g / dL | |  |
| --- | --- | --- | --- | --- | --- |
| 2**-** | Biuret Reagent: |  |  | |  |
|  | Cupric sulfate |  | 6mmol / L | |  |
|  | Sodium potassium tartrate |  | 21mmol / L | |  |
|  | Sodium hydroxide | | 750 mmol / L | |  |
|  | Potassium iodide | |  | 6mmol / L | |

**Procedure**

|  | Blank( ml ) | Standard( ml ) | Sample( ml ) |
| --- | --- | --- | --- |
| Standard | - | 0.025 | - |
| Sample | - | - | 0.025 |
| Reagent 2 | 1.0 | 1.0 | 1.0 |

Mix well, incubate for 10 min. at 37°C. Read the absorbances of the sample (A_sample_) and

standard (A_standard_) against reagent blank at 550 nm. (520 - 570 nm). Color stable for one hour. Linearity up to 10 g / dL.

**Calculation**

$$\mathbf{Protein Concentration (g /dL})=\frac{A sample}{A standard} X 5$$

- - - 1. **Calculation of serum globulin**

according to **Coles [5]**, Serum globulin concentration was calculated by subtracting the serum albumin value from the total serum protein value of the same sample.

- - - 1. **GPT (ALT) Glutamic – Pyruvic Transaminase**

According to **(Reitman and Frankel, 1957)** GPT (ALT) Glutamic – Pyruvic Transaminasewas measured calorimetrically following the instruction defined in the pamphlet

**Principle**

Colorimetric determination of GPT (ALT) activity according to the following reactions:

GPT

Alanine + α-ketoglutarate pyruvate + glutamate

The keto acid pyruvate formed is measured in its derivative form, 2,4- dinitrophenylhydrazone

**Reagents**

| 1. | GPT Buffer substrate:  Phosphate buffer pH 7.5  Alanine  α-Ketoglutarate | 100 mmol/L  200 mmol/L  2 mmol/L |
| --- | --- | --- |
| 2. | Color Reagent:  2,4 dinitrophenylhydrazine | 1 mmol/L |
| 3. | Standard pyruvate | 2 mmol/L |
|  | Additional reagent: ( available on request ) Sodium hydroxide 0.4 N | |

**Procedure**

|  | GPT ( ml ) |
| --- | --- |
| Reagent 1 | 0.5 |
| Incubate for 5 min. at 37°C | |
| Serum | 0.1 |
| Mix and incubate at 37°C for : | Exactly 30 min |
| Reagent 2 | 0.5 |
| Mix. Let stand for 20 min. at room temperature. | |
| NaOH 0.4 N | 5.0 |
| Mix. Wait 5 min. Measure the absorbances at 505 nm (490 – 520 nm) against d. Water using cuvettes 1 cm light path. The color is stable for one hour. Linearity up to 120 units/ ml. | |

**Calculation**

Calculated the number of units/ml of GPT of sample using standard curve

**Standard curve**

Pipette into test tube 1ml:

| Tube No. | 1 | 2 | 3 | 4 | 5 |
| --- | --- | --- | --- | --- | --- |
| D. Water | 0.1 | 0.1 | 0.1 | 0.1 | 0.1 |
| Reagent (1) | 0.5 | 0.45 | 0.4 | 0.35 | 0.3 |
| Pyruvate (R3) | - | 0.05 | 0.1 | 0.15 | 0.2 |
| Reagent (2) | 0.5 | 0.5 | 0.5 | 0.5 | 0.5 |
| Mix. Let stand for 20 min. at room temperature | | | | | |
| NaOH 0.4 N | 5 | 5 | 5 | 5 | 5 |
| Mix. Wait 5 min. Measure as for test at 505 nm | | | | | |
| GPT units/ ml | 0 | 25 | 50 | 83 | 126 |

- - - 1. **GOT (AST) Glutamic – Oxaloacetic Transaminase**

According to **Reitman and Frankel [6]** , GOT (AST) Glutamic – Oxaloacetic Transaminase was measured calorimetrically following the instruction defined in the pamphlet

**Principle**

Colorimetric determination of GOT (AST) activity according to the following reaction

GOT

Aspartate + α-Ketoglutarate oxaloacetate + glutamate

The keto acid oxaloacetate formed is measured in its derivative form, 2,4- initrophenylhydrazone

**Reagents**

| 1. | GOT Buffer substrate: Phosphate buffer pH 7.5  Aspartate  α-Ketoglutarate | 100 mmol/L  100 mmol/L  2 mmol/L |
| --- | --- | --- |
| 2. | Color Reagent:  2,4 dinitrophenylhydrazine | 1 mmol/L |
| 3. | Standard pyruvate | 2 mmol/L |
|  | Additional reagent: ( available on request ) Sodium hydroxide 0.4 N | |

**Procedure**

|  | GOT ( ml ) |
| --- | --- |
| Reagent 1 | 0.5 |
| Incubate for 5 min. at 37°C | |
| Serum | 0.1 |
| Mix and incubate at  37°C for : | exactly  60 min |
| Reagent 2 | 0.5 |
| Mix. Let stand for 20 min. at room temperature. | |
| NaOH 0.4 N | 5.0 |
| Mix. Wait 5 min. Measure the absorbances at 505 nm (490 – 520 nm) against d. Water using cuvettes 1 cm light path. The color is stable for one hour. Linearity up to 150 units/ ml. | |

**Calculation**

Calculated the number of units/ml of GOT of sample using standard curve

**Standard curve**

Pipette into test tube 1ml:

| Tube No. | 1 | 2 | 3 | 4 | 5 |
| --- | --- | --- | --- | --- | --- |
| D. Water | 0.1 | 0.1 | 0.1 | 0.1 | 0.1 |
| Reagent (1) | 0.5 | 0.45 | 0.4 | 0.35 | 0.3 |
| Pyruvate (R3) | - | 0.05 | 0.1 | 0.15 | 0.2 |
| Reagent (2) | 0.5 | 0.5 | 0.5 | 0.5 | 0.5 |
| Mix. Let stand for 20 min. at room temperature | | | | | |
| NaOH 0.4 N | 5 | 5 | 5 | 5 | 5 |
| Mix. Wait 5 min. Measure as for test at 505 nm | | | | | |
| GOT units/ ml | 0 | 22 | 55 | 95 | 150 |

- - - 1. **Alkaline phosphatase - ALP**

According to **Belfield and Goldberg [7]** , Alkaline phosphatase – ALP was measured calorimetrically following the instruction defined in the pamphlet

**Principle**

**Alkaline phosphatase**

Phenyl phosphate phenol + phosphate

**PH 10.0**

The liberated phenol is measured colorimetrically in the presence of 4- aminophenazone and potassium ferricyanide.

**Reagents**

| **1.** | **Standard phenol** | 1.59 mmol / L | |
| --- | --- | --- | --- |
| **2.** | **Buffer – Substrate :** Buffer pH 10.0 Phenyl phosphate | 50  5 | mmol / L mmol / L |
| **3.** | **Enzyme Inhibitor:**  EDTA  4-Aminophenazone | 100  50 | mmol / L mmol / L |
| **4.** | **Color Reagent:**  Potassium ferricyanide | 200 | mmol / L |

**Procedure**

|  | Standard  ( mL ) | Sample  ( mL ) | Blank  ( mL ) |
| --- | --- | --- | --- |
| Reagent 1 | 0.025 | - | - |
| Serum | - | 0.025 | - |
| Reagent 2 | 0.50 | 0.50 | 0.50 |
| Incubate for 20 min. exactly at 37°C, then add: | | | |
| Reagent 3 | 0.25 | 0.25 | 0.25 |
| Mix well, then add : | | | |
| Reagent 4 | 0.25 | 0.25 | 0.25 |

Mix, let stand for 5 min. at room temp. in the dark. Read the absorbances of sample (A_Sample_) and standard (A_standard_) against reagent blank at 510 nm. The color is stable for one hour. The reaction is linear up to 250 IU / L.

**Calculation**

$$\mathbf{Enzyme activity (IU/L)}=\frac{A sample}{A standard} X 75$$

- - - 1. **Urea**

According to **Fawcett and Scott [8]**, urea was measured calorimetrically following the instruction defined in the pamphlet

**Principle:**

The method is based on the following reaction:

Urease

Urea + H_2_O 2 NH_3_ + CO_2_

The ammonium ions formed are measured by the Berthelot reaction. The blue dye indophenol product reaction absorbs light between 530 nm and 560 nm proportional to initial urea concentration.

| **1.** | Standard | 50 mg / dL  (8.3 mmol/L) |
| --- | --- | --- |
| **2.** | Buffer Enzyme:  Phosphate buffer  Urease | 50 mmol / L  >10000 u / L |
| **3.** | Color Reagent: Phenol  odium nitroprusside | 100 mmol / L  0.2 mmol / L |
| **4.** | Alkaline Reagent: Sodiumhydroxide  Sodiumhypochlorite | 150 mmol / L  15mmol / L |

**Reagents**

**Procedure**

|  | Blank (ml) | Standard  (ml) | Sample  (ml) |
| --- | --- | --- | --- |
| Standard | - | 0.01 | - |
| Sample | - | - | 0.01 |
| Reagent 2 | 0.2 | 0.2 | 0.2 |
| Mix, incubate for 5 min, at 37ºC | | | |
| Reagent 3 | 1.0 | 1.0 | 1.0 |
| Reagent 4 | .0 | 1.0 | 1.0 |

Mix, incubate for 10 min. at 37ºC. Measure the absorbance of the sample (A _sample_) and of the standard (A _Standard_) against the blank at 550 nm, (530 – 570 nm). Color stable for 5 hours. Linearity up to 200 mg / dl (33.3 mmol/L) in serum or plasma and 4 g / dl (665 mmol/L) in urine.

**Calculation**

$$urea concentration=\frac{A sample}{A standard} X standard concentration$$

- - - 1. **Creatinine**

According to **Schirmeister et al. [9]** serum creatinine was measured calorimetrically following the instruction defined in the pamphlet

**Principle**:

Creatinine forms a colored complex with picrate in an alkaline medium.

**Reagents**

| 1. | Standard | 2 mg / dL (177 µmol/L) |
| --- | --- | --- |
| 2. | Picric Acid | 20 mmol / L |
| 3. | Sodium hydroxide | 1.2 mmol / L |
|  | Additional reagent: ( available on request ) Trichloroacetic acid ( TCA ) 1.2 mol / L | |

**Deproteinization:**

| Pipette into centrifuge tube : | |
| --- | --- |
| Trichloroacetic acid | 0.5 ml |
| Serum or heparinized plasma | 0.5 ml |

Mix well. Wait for 5 min. Centrifuge for 10 min. at 3000 rpm, then carefully pour the clear supernatant into dry test tube. The supernatant can be stored to seven days at +4 °C

**Procedure**

Working reagent

Mix equal volumes of reagents 2 and 3 immediately before the assay.

|  | Blank  mL | Standard  mL | Serum  mL | Urine  mL |
| --- | --- | --- | --- | --- |
| Dis. H_2_O | 0.25 | - | - | - |
| Reagent 1 | - | 0.25 | - | - |
| TCA | 0.25 | 0.25 | - | 0.25 |
| Serum  supernate | - | - | 0.5 | - |
| Urine diluted | - | - | - | 0.25 |
| Working  Reagent | 0.5 | 0.5 | 0.5 | 0.5 |
| Mix. Incubate 5 min. at 37°C. Measure the absorbances of sample (A_Sample_) and standard (A _Standard_) against the blank at 520 nm. (500 – 550 nm). Linearity up to10 mg / dl in serum or plasma and 300 mg /dl in diluted urine. | | | | |

**Calculation**

$$serum creatinine (mg/dl)=\frac{A sample}{A standard} X2$$

- - - 1. **Uric acid**

According to **Kageyama [10]**, uric acid was measured calorimetrically following the instruction defined in the pamphlet

**Principle:**

Uricase

Uric acid + 2H2O + O Allantoin + CO2 + H2O2

Peroxidase

H2O2 + 3.5, Dichloro-2- hydroxybenzensulphonate + 4-Amino antipyrine H_2_O + HCL + Colored quinoneimine

**Reagent**

| **1.** | **Standard** | 6 mg / dL  (395 µmol/L) |
| --- | --- | --- |
| **2.** | **Chromogen – Buffer**  **Tris buffer** | 50 mmol / L |
|  | 3,5, Dichloro – 2 – hydroxybenzen | |
|  | Sulphonate  Surface – active agent. | 5.0 mmol / L |
| **3.** | **Enzymes:**  Uricase Peroxidase  4 -Aminoantipyrine  Stabilizer and preservative | > 500 IU / L  > 2000 U / L  0.20 mmol /L |

**Procedure**

| Working reagent: Mix equal volumes of reagent 2 and 3 immediately before the assay. | | | |
| --- | --- | --- | --- |
|  | **Blank**  **Ml** | **Standard**  **Ml** | **Sample**  **ml** |
| **Standard** | **-** | **0.05** | **-** |
| **Sample** | **-** | **-** | **0.05** |
| **Working reagent** | **1.0** | **1.0** | **1.0** |

Mix, incubate for 10 min. at 37°C. Read the absorbances of the sample (A _Sample_) and the standard (A_Standard_) against blank, at 510 nm. (490 - 550). Color is stable for 30 min.

Linearity up to 30 mg / dL**.**

**Calculation**

$$uric acid in serum =\frac{A sample}{A standard} Xstandard concentration$$

**Haematological examination**

Haematological profile involved total counts (TC) of the RBC (red blood corpuscles) and WBC (white blood corpuscles), differential counts (DC) of the WBC, HCT (hematocrit), and Hb (hemoglobin) concentration. The hematological parameters were estimated using the techniques described by **Ritchie et al. [11].** After blood collection, a blood film was prepared from blood containing EDTA. A slide and a rectangular coverglass (24 mm x 50 mm) were used to make a film on a slide. Wright's stain was used to stain air-dried blood films. The leftovers of the blood sample after making a blood film were used to obtain a hematocrit or packed cell volume (PCV), hemoglobin concentration and cell count. The Hct was occurred by centrifuging a microhematocrit tube full of blood at 12,000 G for five minutes. By using the manual cyanomethemoglobin method after centrifugation and removal of free red cell nuclei and membrane debris, hemoglobin concentration was measured calorimetrically. Red blood cell (RBCs) count was obtained manually by Natt and Herrick's method. The latter process requires the preparation of a methyl violet 2B diluent. This solution and a dilution pipette were used to make a 1: 200 dilution of blood. After mixing, the diluted blood was drained into a Neubauerruled hemacytometer and the cells were allowed to settle on the surface for 5 minutes before counting. Red blood cells were counted using one of the four corner squares and the central square of the large central primary square on the hemocytometer. The number of red blood cells counted was multiplied by 10,000 to determine the number of red blood cells per microliter of blood. Appropriate secondary squares are counted on each grid and the counts are averaged.

The number of avian white blood cells (WBC) was obtained using a manual technique according to **Natt and Herrick [12]** because the presence of nucleated red blood cells and platelets interferes with the number of white blood cells using the electron cell counter. Direct white blood cell counts using the Natt and Herrick's method. The DC of WBC was performed using a blood cell counter and WBC pipette, where the numbers of neutrophils, eosinophils, lymphocytes, and monocytes were recorded per 100 cells**.**

**Immunohistochemical procedures**

**Aim:** The aim of Immunohistochemistry (IHC) was the detection of antigens either cellular or even hapten in cells through the binding of antibodies to specific antigens in biological tissues.

**Principle:** The antibody-antigen binding could be seen by enzymes, such as Horseradish Peroxidase (HRP) or Alkaline Phosphatase (AP) that are commonly used to catalyze a color-producing reaction. IHC was currently used in multiple research and clinical laboratories. IHC had importantly visualized the distribution and localization of specific cellular components within cells.

**Steps (Saber et al. [13])**

1. Liver samples of different birds were directly trimmed and immersed in neutral buffer formalin.
2. Fixation of samples was done for 4 days.
3. Routine histological techniques were done to all the samples including the previous steps used in histological sections such as dehydration, clearance, embedding and cutting by microtome.
4. Tissue ribbon was mounted on positive charged slides to avoid their separation during the autoclaving step.
5. Then slides were rehydrated through immersion in xylene, alcohols and then water.
6. Antigen retrieval step which aimed to remove methylene bridges on the protein caused by formalin, therefore it is too essential to unmask the antigen epitopes in order to allow the antibodies to bind with it. This step was done by immersion of the samples within in a solution of 0.05 M citrate buffer, pH 6.8.
7. Inhibition of the endogenous cellular enzymes to avoid nonspecific binding of horseradish peroxidase (HRP) or alkaline-phosphatase (AP). Thus samples were put in 0.3 % H_2_O_2_ and protein block with sera of the animal spp of the secondary antibody at room temperature for 30 min.
8. After that, slides were incubated with the specific antibody with specific dilution anti-NF-ĸB P65, Santa cruz, Cat# (F-6): sc-8008, 1:100 dilution).
9. The slides were rinsed with PBS three times for 10 of each.
10. The slides were incubated with a goat anti-rabbit secondary antibody (cat. no. K4003, EnVision+™ System Horseradish Peroxidase Labelled Polymer; Dako) for 30 min at room temperature.
11. Slides were visualized with DAB kit (3,3’-Diaminobenzidine) and eventually stained with Mayer's haematoxylin as a counterstain.
12. The staining index was assessed and presented as the mean of positive cells in 8 high power fields as in caspace 3 and bax antibodies, and data were expressed as the percent of positive area±SE.

Reference

1. Shotwell OL, Burg WR, Diller T. Thin layer chromatographic determination of aflatoxin in corn dust. J Assoc Off Anal Chem. 1981 Sep 1;64(5):1060-3.
2. AOAC. 1980. Natural Poisons, 13th ed. Association of Official Analytical Chemists, Washington, DC., USA.
3. Doumas BT, Ard Watson W, Biggs HG. Albumin standards and the measurement of serum albumin with bromcresol green. Clinica Chimica Acta. 1971; 31(1): 87–96.
4. Gornall AG, Bardawill CJ, David MM. Determination of serum proteins by means of the biuret reaction. J Biol Chem 1949 Feb 1;177(2):751-66.
5. Coles EH. Veterinary clinical pathology. 1974 (Issue Ed. 2). WB Saunders.
6. Reitmen S, Frankel S. A colorimetric method for the determination of serum glutamate oxaloacetate and serum glutamate pyruvate transminase. Am J Clin Pathol. 1957;28 (1):56.
7. Beliefield A, Goldberg DM. Estimation of serum alkaline phosphates. Enzyme. 1971;12: 561–573.
8. Fawcett JK, Scott JE. Colorimetric determination of urea. An J Clin Path. B. 1960; 13: 156–159.
9. Schirmeister J, Willmann H, Kiefer H. Plasma creatinine as rough indicator of renal function. Deutsche medizinische Wochenschrift (1946). 1964 May 22;89:1018-23.
10. Kageyama N. A direct colorimetric determination of uric acid in serum and urine with uricase-catalase system. Clinica Chimica Acta. 1971; 31(2), 421–426.
11. Ritchie BW, Harrison JG, Harrison RL. Avian medicine: principle and application. Winger’s Publishing. Inc., Florida, FL 1994.
12. Natt MP, Herrick CA. A new blood diluent for counting the erythrocytes and leucocytes of the chicken. Poul Sci. 1952 Jul 1;31(4):735-8.
13. Saber S, Khalil RM, Abdo WS, Nassif D, El-Ahwany E. Olmesartan ameliorates chemically-induced ulcerative colitis in rats via modulating NFκB and Nrf-2/HO-1 signaling crosstalk. Toxicol Appl Pharmacol. 2019 Feb 1;364:120-32.
